# Distinctive Formation of Volatile Compounds in Fermented Rice Inoculated by Different Molds, Yeasts, and Lactic Acid Bacteria

**DOI:** 10.3390/molecules24112123

**Published:** 2019-06-05

**Authors:** Min Kyung Park, Young-Suk Kim

**Affiliations:** Department of Food Science and Engineering, Ewha Womans University, Seoul 03760, Korea; carrot0412@gmail.com

**Keywords:** fermented rice, volatile compounds, microbial effects, volatile metabolic pathways

## Abstract

Rice has been fermented to enhance its application in some foods. Although various microbes are involved in rice fermentation, their roles in the formation of volatile compounds, which are important to the characteristics of fermented rice, are not clear. In this study, diverse approaches, such as partial least squares-discriminant analysis (PLS-DA), metabolic pathway-based volatile compound formations, and correlation analysis between volatile compounds and microbes were applied to compare metabolic characteristics according to each microbe and determine microbe-specific metabolites in fermented rice inoculated by molds, yeasts, and lactic acid bacteria. Metabolic changes were relatively more activated in fermented rice inoculated by molds compared to other microbes. Volatile compound profiles were significantly changed depending on each microbe as well as the group of microbes. Regarding some metabolic pathways, such as carbohydrates, amino acids, and fatty acids, it could be observed that certain formation pathways of volatile compounds were closely linked with the type of microbes. Also, some volatile compounds were strongly correlated to specific microbes; for example, branched-chain volatiles were closely link to *Aspergillus oryzae*, while *Lactobacillus plantarum* had strong relationship with acetic acid in fermented rice. This study can provide an insight into the effects of fermentative microbes on the formation of volatile compounds in rice fermentation.

## 1. Introduction

Fermented rice products, such as alcoholic beverages (*makgeolli*, *sake*, and Chinese rice wine), fermented soybean pastes (*deonjang* and *miso*), and rice vinegar [1,2,3], have been consumed mainly due to their characteristic odors and tastes. During rice fermentation, fermentative microbes degrade macromolecules of food substrates (e.g., proteins, carbohydrates, and lipids) via diverse enzymatic metabolic pathways, providing a precursor pool of amino acids, sugars, and fatty acids that can participate in further biological and chemical reactions, so as to produce volatile compounds related to the characteristic tastes and odors [4]. Regarding previous studies on the main volatile compounds in fermented rice, Xiao et al. [5] reported the major volatile compounds in Chinese rice vinegars were furfural, acetic acid, ethyl acetate, 3-hydroxy-2-butanone, 3-methyl-1-butanol, isopentyl acetate, bezaldehyde, and phenylethyl alcohol. Son et al. [6] also compared the volatile compounds profiles in *makgeolli* fermented by *Asepergillus oryzae* and *Saccharomycopsis fibuligera*. Fusel alcohols, acetate esters, and ethyl esters were considered as the main volatile compounds of *makgeolli*.

On the other hand, the enzymes of microbes are specific to perform specialized catalytic reactions, forming different metabolites according to the type of microbes despite being exposed to identical culture conditions [7]. Diverse microbes (e.g., *Aspergillus* spp., *Saccharomyces cerevisiae*, non-*Saccharomyces* yeasts, and *Lactobacillus* spp.) have been commonly used as microbial starters in fermented rice products, such as *makgeolli* and rice vinegar [8,9]. In particular, molds such as *Aspergillus* spp. and *Rhizopus* spp. are preferentially used in rice fermentation and represented as an initial fermentative microbe due to their higher hydrolytic enzymes, such as amylase, protease, and glucoamylase [10,11]. On the other hand, yeasts, especially, *Saccharomyces cerevisiae*, are known for generating some volatile compounds, such as fusel alcohols and esters, in wine making, baking, and brewing [12]. In addition, lactic acid bacteria are also known as the characteristic fermentative microbes for producing organic acids, including acetic acid and lactic acid, so that acidification occurs in fermented foods [13]. The fermentative microbes mentioned above can also significantly affect the generation of volatile compounds in fermented rice. Thus, in order to investigate the quality of fermented rice, studies on the formation of characteristic volatile compounds based on microbial species should be carried out.

Although some studies on the formation of volatile compounds according to various microbes have been performed in rice fermentation [6,14], there is little research on the comprehensive determination of specific volatile compounds related to the range of microbes which are responsible for various rice fermentations. In this study, some approaches, such as partial least squares-discriminant analysis (PLS-DA) and metabolic pathway-based and correlation analysis, were applied to identify the characteristic patterns of volatile compound profiles distinguished from other species in fermented rice. The aim of this study was to compare the profiles of induced volatile compounds in fermented rice inoculated by diverse molds, yeasts, and lactic acid bacteria according to fermentative microbes. Solid phase microextraction-gas chromatography-mass spectrometry (SPME-GC-MS) was applied to obtain the volatile compound profiles of steamed and non-inoculated (control) or fermented rice. The mass spectrometry-based data sets were combined with correlation analysis to identify the differences in volatile metabolite profiles according to each microbe and to determine the microbe-specific volatile compounds in rice fermentation.

## 2. Results and Discussion

### 2.1. PLS-DA for Volatile Metabolite Profiles in Fermented Rice

**Figure 1:**
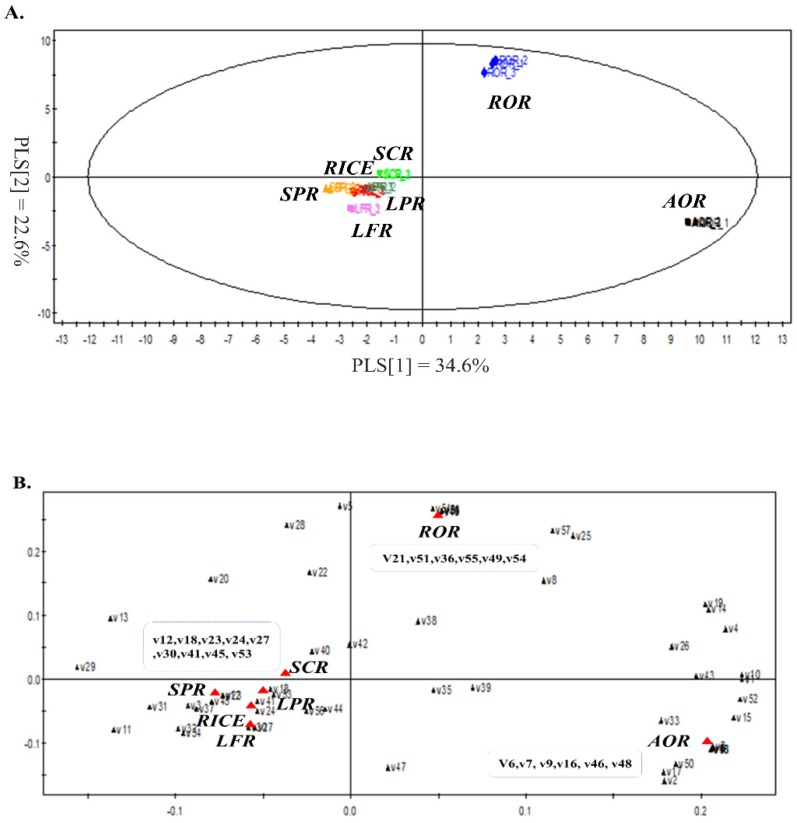
Partial least squares-discriminant analysis (PLS-DA) score plot for volatile compounds profiles in fermented rice inoculated by different microbes. (**A**) Score plot, (**B**) loading plot. AOR = *Aspergillus oryzae*, ROR = *Rhyzopus oryzae*, SCR = *Sacharomyces cerevisiae*, SFR = *Saccharomycopsis fibuligera*, LFR = *Lactobacillus fermentum*, LPR = *Lactobacillus plantarum*, RICE = controls (non-fermented rice).

PLS-DA was performed to evaluate the differences among volatile compound profiles obtained from GC-MS data for different fermentation microbes (molds, yeasts, and lactic acid bacteria). The classification of different fermented samples and controls (RICE, non-inoculated and steamed rice), and also discriminant volatile compounds, are shown in Figure 1. The PLS-DA model explained 57.2% of total variance, such as 34.6% PLS [1] and 22.6% PLS [2]. The model was robust and predictable. The parameters of the cross-validation modeling for the third PLS component were R^2^Y = 0.996, and Q^2^Y = 0.997. Permutation testing (50 iterations) was also performed in order to further validate the model, after which R^2^ = 0.131 and Q^2^ = −0.753. 

In a score plot of PLS-DA, AOR (fermented by *Aspergillus oryzae*) and ROR (*Rhyzopus oryzae*) were clearly separated from SCR (*Sacharomyces cerevisiae*), SFR (*Saccharomycopsis fibuligera*), LFR (*Lactobacillus fermentum*), LPR (*Lactobacillus plantarum*), and control sample (RICE, non-fermented rice), and SCR, SFR, LFR, and LPR were closely placed near RICE. It can be assumed that *A. oryzae* and *R. oryzae* were more active in the changes of volatile compounds during rice fermentation than other microbes.

Table 1 lists the distinct volatile compounds from each microbe. A total of 21 significant variables contributing to discriminate samples (*p*-value >0.1 or <−0.1) were selected on the basis of a threshold of 1.0 on the variable importance in the projection (VIP) test. Some volatile metabolites derived from amino acids, such as 2-methylbutanal, 3-methylbutanal, ethyl 2-methylbutanoate, 2-methylpropanoic acid, and 2-phenylacetaldehyde, were highly correlated to AOR, while 3-methylbutyl acetate, 5-ethyloxolan-2-one, 3-ethoxypropan-1-ol, ethyl tetradecanoate, 1-phenylethanone, and 2-phenylethanol, which are mainly derived from amino acids and fatty acids, were representative of ROR. In addition, volatile metabolites derived from fatty acids and butanediol fermentation were major discriminant volatile compounds in other samples, such as RICE, SCR, SFR, LFR, and LPR.

Table 2 shows the identified volatile compounds with their relative peak areas and retention indices (RI). A total of 57 volatile compounds, such as eight carbohydrate-derived, 20 amino acid-derived, and 29 fatty acid-derived volatile compounds, were found in all samples.

During rice fermentation, the generation of volatile compounds varied according to each microbe in this study. In addition, we observed that the total contents of volatile compounds were much higher in AOR and ROR compared to other samples. The major volatile compounds generated by AOR were ethanol, 3-methylbutan-1-ol, 3-methylbutanal, and 2-methylbutanal, while 3-methylbutan-1-ol, ethanol, and 2-methylpropan-1-ol were the major volatile compounds in ROR. In addition, the contents of ethanol and 3-methylbutan-1-ol were higher than those of other volatile compounds in SCR, while butan-2,3-diol, butane-2,3-dione, and 3-methyl-butan-1-ol were the predominant volatile compounds in SFR. The level of 3-hydroxy-2-butanone was the most abundant in LFR. On the other hand, acetic acid and 3-hydroxy-2-butanone were the main metabolites of LPR, but overall formation of volatile compounds was weakly generated compared to other fermented samples. These results indicated that certain volatile compounds were generated at high levels, specific to each microbe. For example, the level of 3-methylbutan-1-ol in mold and yeast fermentation (AOR, ROR, SCR, and SFR) was higher, while that of 3-hydroxybutan-2-one was superior in lactic acid bacteria fermentation (LFR and LPR). In the following section, we compare the formation of volatile compounds based on the major metabolic processes, such as carbohydrate, amino acid, and fatty acid metabolism, to determine the metabolic characteristics of each microbe. 

### 2.2. Volatile Compounds Based on Metabolic Pathways in Fermented Rice According to Each Microbe

**Figure 2:**
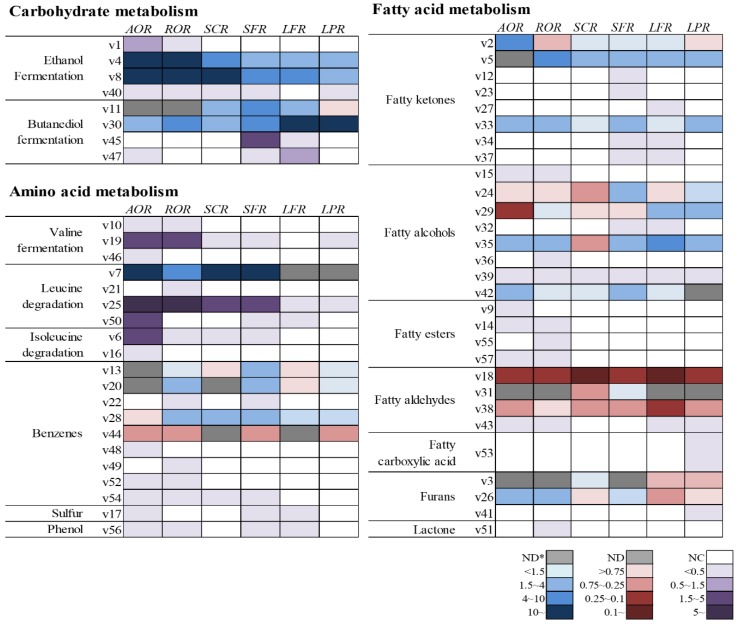
Metabolic pathway-based volatile compound expressions of fermented rice. The fold change of each volatile compound was calculated by comparing to control (RICE): ND; not detected, NC; not changed. Each square represents the fold change (log_2_ level) against the control based on the color gradient as shown in the legend (blue; positive, red; negative, and gray; not detected). The formation of volatile compounds which were not detected in the control but were newly produced during fermentation were marked separately (purple; newly detected, white; not detected). Sample abbreviations are shown below; AOR (fermented by *Aspergillus oryzae*), ROR (*Rhyzopus oryzae*), SCR (*Sacharomyces cerevisiae*), SFR (*Saccharomycopsis fibuligera*), LFR (*Lactobacillus fermentum*), LPR (*Lactobacillus plantarum*), and RICE (non-fermented rice).

The formation of volatile compounds in fermented rice are visually represented by heatmaps in Figure 2. The fold changes of volatile compounds, which were calculated compared to the control (RICE), were classified by three main metabolic pathways, including carbohydrate, amino acid, and fatty acid metabolism (Table S1). Heatmaps were clustered by defined pathways provided from kyoto encyclopedia of genes and genomes (KEGG) pathways and previous studies [15,16,17]. The tendency to increase or decrease compared to the control was shown in five stages (blue; positive, red; negative, and gray; not detected), while the formation of volatile compounds which were not detected in the control but were newly produced during fermentation, were marked separately (purple; newly detected, white; not detected).

As shown in Figure 2, the heatmap involved in carbohydrate metabolism was more activated compared to other forms of metabolism during rice fermentation. Carbohydrates were closely linked to the formation of some volatile compounds, such as ethanol fermentation and butanediol fermentation. Ethanol originates from fermentative breakdown of glucose and other hexose via the Embden–Meyerhof–Parnas (EMP) pathway and some other pathways with decarboxylase and dehydrogenase [18]. By-products of ethanol fermentation, including acetic acid, ethyl acetate, and acetaldehyde, were found in this study. In particular, acetaldehyde and acetic acid were detected only in the fermented samples, with the exception of RICE (non-fermented). The increase of acetaldehyde was higher in AOR, while that of acetic acid was superior in LPR compared to other samples. On the other hand, the overall by-products of ethanol fermentation were more actively generated in AOR, ROR, and SCR. In particular, ethanol and ethyl acetate were generated at amounts approximately 200- and 25-fold higher, respectively, than those in the control.

The three-stage butanediol fermentation pathway begins with the combination of two molecules of pyruvate from glycolysis to form α-acetolactate by α-acetolactate synthase (α-ALS). α-Acetolactate is then decarboxylated to butane-2,3-dione (diacetyl) and 3-hydroxybutan-2-one (acetoin) by α-acetolactate decarboxylase (α-ALD) and, finally, 2,3-butanediol can be formed by the reduction of acetoin by 2,3-butanediol dehydrogenase (BDH) [19]. In this study, AOR, ROR, LFR, and LPR showed a higher generation of acetoin than other metabolites. In particular, lactic acid bacteria such as LFS and LPS were strongly associated with a higher production of acetoin, consistent with the results of a previous study [20]. On the other hand, the levels of diacetyl and acetoin in SCS and SPS were similar, while the formation of butan-2,3-diol was significantly increased in SPS.

Amino acid metabolism can be involved in the generation of some volatile compounds, such as sulfur-containing volatiles, branched-chain volatiles, and aromatic volatiles [21,22]. They come from the degradation of specific amino acids, such as sulfur-containing (methionine and cysteine), branched-chain (leucine, valine, and isoleucine), and aromatic ones (phenylalanine, tyrosine, and tryptophan). In this study, branched-chain volatiles, except for 3-methylbutan-1-ol, were detected only in the fermented samples. In particular, levels of 2-methylbutanal, 3-methylbutanal, 3-methylbutan-1-ol, and 3-methylbutanoic acid were greatly increased in AOR, while much lower levels of these compounds were produced by LPR and LFR compared to other samples. This result might be due to the absence of certain enzymes, such as transaminase, decarboxylase, and hydrogenase, or weaker activities of these enzymes in lactic acid bacteria than what is found in other microbes. 

With regard to products of benzenes, the fold change of benzaldehyde was decreased or not found in all samples studied. It could be assumed that benzaldehyde can be converted to other benzenes, such as styrene, 2-phenylethanol, ethyl 2-phenylacetate, 1-phenylethanone, toluene, and ethylbenzene. In particular, 2-phenylethanol with a rose-like odor is commonly found in alcoholic beverages, such as makgeolli and sake [6,23]. 2-Phenylethanol can be converted to 2-phenylacetaldehyde and ethyl 2-phenylacetate by phenylacetaldehyde reductase and alcohol acetyltransferase, respectively [24]. In this study, the levels of 2-phenylacetaldehyde were greater in AOR than those of benzoic compounds, while that of 2-phenylethanol was higher in ROR, SCR and SFR. Regarding other benzoic volatile compounds, 4-ethenyl-2-methoxyphenol, 1-phenylethanone and styrene can be converted from cinnamic acid which is derived from phenylalanine degradation [25].

A compound called 4-ethenyl-2-methoxyphenol, which is known as the main volatile compound of rice and has a smoky odor, was the only phenolic volatile compound found in this study [26]. The formation of 4-ethenyl-2-methoxyphenol is characterized by a two-step process. Firstly, cinnmic acid is converted to hydroxycinnamic acid by esterase. Then, cinnamate decarboxylase catalyzes its transformation to 4-ethenyl-2-methoxyphenol [27]. In ROS, the formation of these benzoic volatiles was more activated compared to other samples, while that of styrene was decreased and 1-phenylethanone was not detected in AOR. The only sulfur-containing volatile compound in this study, (methyldisulfanyl)methane, could be produced from methionine. Methionine is degraded to methanethiol by methionine-γ-lyase, and then oxidized to (methyldisulfanyl)methane [22]. This compound, which had been reported as an off-odorant in yakju (Korean rice liquor) [28], was detected only in AOR, SFR, and LFR in this study.

During fermentation, lipids can be hydrolyzed to glycerol and fatty acids by lipase. The fatty acid metabolic pathway includes both fatty acids and their derivatives. There are various volatile compounds derived from fatty acids, including furans, lactones, and volatile compounds derived from fatty acids (aldehydes, acids, ketones, and esters). β-Oxidation, which is a main fatty acid breakdown pathway, converts fatty acids into two carbon segments (acetyl-CoA), then, further enzymatic activities, such as decarboxylation, oxidation, reduction, and so on, catalyze the formation of aldehydes, alcohols, ketones, acids, and esters [29]. Esters derived from fatty acids, such as ethyl propanoante, ethyl butanoate, ethyl tetradecanoate, and ethyl hexadecanoate, were only detected in AOR and ROR. These volatile compounds can be formed by esterification, combining fatty acids and ethanol, which is catalyzed by esterase [29]. A marked content of ethanol was observed in AOR, ROR, and SCR, while fatty acid esters were not found in SCR. It might be assumed that the different activity of esterase of the individual microbes could affect the formation of fatty acid esters. In addition, higher numbers of ketones were detected in SFR than in other samples in this study. 

Lactones are mainly produced from unsaturated fatty acids, such as linoleic acid and oleic acid [30], and significantly contribute to the aroma of fermented rice products, such as rice beer and Chinese rice wine, as fruity and floral odor notes [31,32]. An important odor component of peach [33], 5-ethyloxolan-2-one (γ-hexalactone), was only detected in ROR.

### 2.3. Microbe-Specific Volatile Compounds in Fermented Rice

Fermented rice is enjoyed due to its characteristic aroma and taste [34] as well as its health benefits [35], which are not present in raw rice. In this study, a correlation analysis was performed to determine the microbe-specific volatile compounds involved in the flavor properties of fermented rice. Volatile compounds related to each microbe were selected using a threshold of 0.7 coefficient (r) (*p*-value < 0.05) (Table 3).

Relatively more diverse volatile compounds were linked to AOR compared to other samples. In particular, branched-chain volatiles, such as ethyl 2-methylbutanonoate, 2-methylpropanoic acid, 2-methylbutanal, 3-methylbutanal, 3-methylbutanoic acid, and ethyl 2-methylpropanoate, were more highly associated with AOR compared to other microbes. It can be explained that the activities of certain enzymes, such as protease, branched-chain amino acid aminotransferase, and so on, in *A. oryzae* were higher than in other microbes.

On the other hand, some volatile compounds, such as esters derived from fatty acids (ethyl tetradecanoate and ethyl hexadecanoate) and lactone (5-ethyloxolan-2-one), were closely related with ROR. *R. oryzae* might have superior activities of some enzymes related to the formation of esters and lactones, such as lipase, esterase, lactonizing enzymes, and so on.

Regarding yeast fermentation, SFR was linked to a greater number of distinctive volatile compounds that can be distinguished from other fermented rice compared to SCR. Some ketones derived from fatty acids (3-methylpentan-2-one and 5-methylhexan-2-one) and volatiles derived from butanediol fermentation (diacetyl and butane-2,3-diol) were highly involved in SFR, while SCR was linked to only oxolan. 

In this study, the content of ethanol induced by *S. cerevisiae*, which is the most commonly employed yeast for ethanol production [36], was highest, but other microbes, such as *A. oryzae* and *R. oryzae*, could also contribute to the formation of ethanol.

Some volatile compounds from lactic acid bacteria fermentation, such as acetoin (a yogurt-like odorant) and 4-ethenyl-2-methoxyphenol (a smoky odorant), were closely linked with LFR, while acids, such as hexanoic acid and acetic acid, were positively associated with LPR. 

## 3. Material and Methods

### 3.1. Chemicals and Reagents

HPLC-grade methanol and water were obtained from J. T. Baker (Phillipsburg, NJ, USA) and sodium chloride was purchased from Sigma-Aldrich (St. Louis, MO, USA).

### 3.2. Sample Preparation

Fermentative microbes isolated from fermented foods, such as *Aspergillus oryzae* KACC46102 (AOR), *Rhizopus oryzae* KACC40242 (ROR), *Saccharomyces cerevisiae* KACC30008 (SCR), *Saccharomycopsis fibuligera* KACC47772 (SFR), *Lactobacillus fermentum* KACC 15,736 (LFR), and *Lactobacillus plantarum* KACC 124,401 (LPR), were supported from the Korean Agricultural Culture Collection (KACC, Jeonju-si, Jeollabuk-do, Korea).

Rice (*Oryza sativa* L.) was soaked in water for 16 h and cooked at 121 °C for 15 min before inoculating with 1 × 10^5^ colony forming units (CFU)/g of microorganisms. Then, they were incubated at 30 °C for 48 h. They were kept in a deep freezer (below −70 °C) before analysis.

### 3.3. Volatile Compounds Analysis

The samples were kept in liquid nitrogen for 1 min, before grinding for 60 s using a miller (7011S, Waring Commercial, Stamford, CT, USA). Samples (2.5 g) with 5 mL saturated sodium chloride solution were put into a screw vial with screw cap (Ultraclean 18 mm, Agilent technologies, Santa Clara, CA, USA). Then, 1 μL of an internal standard (L-borneol (Sigma-Aldrich) 100 μg/mL, in methanol) was added into the vial for quantifying the volatile metabolites. Solid phase microextraction (SPME) was applied to extract volatile compounds in this study. It was maintained at 30 °C for 30 min for an equilibrium state. Then, volatile compounds were adsorbed for 30 min onto divinylbenzene/carboxen/polydimethylsiloxane (DVB/CAR/PDMS, 75 μm, Supelco, Bellefonte, PA, USA) coated SPME fiber that was inserted 20 mm into the sealed vial.

Gas chromatography (GC-MS) analysis was performed using a 7890A series gas chromatograph (Agilent Technologies), an CTC-PAL auto sampler (GC sampler 80, Agilent Technologies), and a 5975C mass selective detector (Agilent Technologies) equipped with a DB-WAX column (30 m length × 0.25 mm i.d. × 25 μm film thickness, J&W Scientific, Folsom, CA, USA). Oven temperature was initially held at 40 °C for 6 min and ramped to 82 °C at a rate of 6 °C/min, and then raised to 200 °C (1 min) at 4 °C/min. Helium, a carrier gas, constantly flowed at 0.8 mL/min. Injector and detector temperatures were 230 °C and 250 °C, respectively. The mass spectral data were obtained at 70 eV in electron ionization (EI), with a mass scan range of 35–350 amu at a rate of 4.5 scans/s. The identification of metabolites was positively confirmed by comparing mass spectral data and retention times to those of authentic standard compounds. Otherwise, they were identified with the basis of their mass spectral database (NIST08 and Wiley9n.1) and retention index (RI) values using the national institute of standards and technology (NIST) Chemistry Webbook. The contents of volatile compounds were calculated by comparing their peak areas to those of an internal standard compound.

### 3.4. Statistical Analysis

Analysis of variance (ANOVA) and correlation analysis were accomplished using IMB SPSS Statistics for Windows, version 25.0 (IBM Corp., Armonk, NY, USA) to evaluate statistical change and difference of volatile compound formations in each sample. The result of Duncan’s multi-range test was presented at the significant different level (*p* ≤ 0.05). A partial least squares-discriminant analysis (PLS-DA) was performed to discriminate samples based on the volatile compound profiles using SIMCA-P software (version 11.0, Umetrics, Umea, Sweden).

## 4. Conclusions

We compared the volatile compound profiles and the microbes that produced these compounds during rice fermentation. Diverse approaches, such as multivariable statistical analysis (PLS-DA), comparative analysis based on main pathways related to generate volatile compounds, and correlation analysis between volatile compounds and each microbe, were undertaken to find the key volatile compounds for discrimination of fermented rice samples inoculated by different microbes. In this study, 46 volatile compounds were classified by metabolisms strongly involved in the formation of volatile compounds, regardless of the type of microbes. Some findings corresponded to those of previous studies. For example, *S. cerevisiae* is well known as a higher producer of ethanol and fusel alcohols. In addition, 2-methylbutanal and 3-methylbutanal are major fermentative compounds, induced from fungi, such as *A. oryzae* [16], while lactic acid bacteria are superior for generating organic acids, including acetic acid, and butanediol fermentation [37]. However, we were able to investigate the characteristic features of volatile compound profiles in rice fermentation according to each microbe. The new findings that were revealed in this study showed that *A. oryzae* was highly linked with the formation of volatiles derived from amino acids, while *R. oryzae* was superior for generating volatiles derived from fatty acids. In addition, ester formation was closely linked with *R. oryzae* as well as yeasts, which are typical microbial starters for the production of esters [38]. In conclusion, these results can help in the rational selection of fermentative microbial starters in fermented rice to obtain the proper sensory qualities.

## Figures and Tables

**Table 1:** Discriminant volatile compounds of different samples.

No. ^1^	Volatile Compound	Sample ^2^
v6	2-Methylbutanal	*AOR*
v7	3-Methylbutanal	
v9	Ethyl propanoate	
v16	Ethyl 2-methylbutanoate	
v46	2-Methylpropanoic acid	
v48	2-Phenylacetaldehyde	
v21	3-Methylbutyl acetate	*ROR*
v51	5-Ethyloxolan-2-one	
v36	3-Ethoxypropan-1-ol	
v55	Ethyl tetradecanoate	
v49	1-Phenylethanone	
v54	2-Phenylethanol	
v12	3-Methylpentan-2-one	*RICE/SCR/SPR/LFR/LPR*
v18	Hexanal	
v23	5-Methylhexan-2-one	
v24	Butan-1-ol	
v27	3-Hydroxy-3-methylbutan-2-one	
v30	3-Hydroxybutan-2-one	
v41	Furan-2-carbaldehyde	
v45	Butane-2,3-diol	
v53	Hexanoic acid	

^1^ Numbered as in the order of retention indices (RI). ^2^ All abbreviations are shown below; AOR (fermented by *Aspergillus oryzae*), ROR (*Rhyzopus oryzae*), SCR (*Sacharomyces cerevisiae*), SFR (*Saccharomycopsis fibuligera*), LFR (*Lactobacillus fermentum*), LPR (*Lactobacillus plantarum*), and RICE (non-fermented rice).

**Table 2:** Volatile compounds identified in fermented rice inoculated by different microbes.

No. ^1^	Volatile Compound	RI ^2^	Relative Peak Area (Mean ± SD) ^3^							ID ^4^
			RICE ^5^	AOR	ROR	SCR	SFR	LFR	LPR	
***Carbohydrate metabolism***										
***Ethanol fermentation***										
v1	Acetaldehyde	<800	ND ^6^a ^7^	0.723 ± 0.009c	0.300 ± 0.006b	NDa	NDa	NDa	NDa	B
v4	Ethyl acetate	885	0.013 ± 0.002a	0.438 ± 0.014f	0.337 ± 0.007e	0.092 ± 0.006d	0.025 ± 0.003ab	0.052 ± 0.005c	0.031 ± 0.002b	A
v8	Ethanol	931	0.080 ± 0.007a	19.811 ± 0.558b	31.903 ± 0.914c	33.447 ± 1.427d	0.466 ± 0.021a	0.700 ± 0.021a	0.167 ± 0.003a	A
v40	Acetic acid	1454	NDa	0.058 ± 0.002c	0.152 ± 0.005e	0.038 ± 0.002b	0.096 ± 0.006d	NDa	0.485 ± 0.014f	A
***Butanediol fermentation***										
v11	Butane-2,3-dione	971	0.187 ± 0.010b	NDa	NDa	0.487 ± 0.017c	1.259 ± 0.055e	0.719 ± 0.022d	0.165 ± 0.003b	A
v30	3-Hydroxybutan-2-one	1279	0.037 ± 0.002a	0.069 ± 0.002ab	0.270 ± 0.002b	0.107 ± 0.004ab	0.265 ± 0.012b	12.172 ± 0.310d	0.781 ± 0.024c	A
v45	Butane-2,3-diol	1542	NDa	NDa	NDa	NDa	1.657 ± 0.073c	0.163 ± 0.008b	NDa	A
v47	Butane-1,3-diol	1579	NDa	0.257 ± 0.005c	NDa	NDa	0.195 ± 0.010b	0.507 ± 0.016d	NDa	A
***Amino acid metabolism***										
***Valine degradation***										
v6	2-Methylbutanal	909	NDa	1.485 ± 0.040c	0.014 ± 0.003a	0.070 ± 0.006b	0.011 ± 0.000a	NDa	NDa	A
v16	Ethyl 2-methylbutanoate	1050	NDa	0.110 ± 0.002b	NDa	NDa	NDa	NDa	NDa	A
***Leucine degradation***										
v7	3-Methylbutanal	913	0.011 ± 0.002a	3.824 ± 0.106d	0.108 ± 0.001b	0.237 ± 0.008c	0.125 ± 0.007b	NDa	NDa	A
v21	3-Methylbutyl acetate	1119	NDa	NDa	0.042 ± 0.002b	NDa	NDa	NDa	NDa	A
v25	3-Methylbutan-1-ol	1211	NDa	14.935 ± 0.420c	39.594 ± 1.129d	1.298 ± 0.055b	1.03 ± 0.045b	0.036 ± 0.005a	0.080 ± 0.001a	A
v50	3-Methylbutanoic acid	1683	NDa	1.314 ± 0.035d	NDa	NDa	0.324 ± 0.015c	0.191 ± 0.008b	NDa	A
***Isoleucine degradation***										
v10	Ethyl 2-methylpropanoate	959	NDa	0.462 ± 0.011c	0.204 ± 0.004b	NDa	NDa	NDa	NDa	A
v19	2-Methylpropan-1-ol	1100	NDa	2.439 ± 0.067d	2.359 ± 0.059c	0.216 ± 0.009b	0.032 ± 0.004a	NDa	0.026 ± 0.002a	A
v46	2-Methylpropanoic acid	1575	NDa	0.368 ± 0.008b	NDa	NDa	NDa	NDa	NDa	A
***Benzenes***										
v13	Toluene	1033	0.102 ± 0.019c	NDa	0.146 ± 0.005d	0.082 ± 0.003b	0.260 ± 0.012e	0.082 ± 0.006b	0.110 ± 0.001c	A
v20	Ethylbenzene	1115	0.027 ± 0.002bc	NDa	0.064 ± 0.007d	NDa	0.071 ± 0.005d	0.024 ± 0.004b	0.032 ± 0.002c	A
v22	1,4-Xylene	1130	NDa	NDa	0.046 ± 0.002b	NDa	0.053 ± 0.005c	NDa	NDa	A
v28	Styrene	1250	0.074 ± 0.004a	0.068 ± 0.002a	0.190 ± 0.004f	0.145 ± 0.006e	0.136 ± 0.007d	0.088 ± 0.006b	0.106 ± 0.001c	A
v44	Benzaldehyde	1517	0.178 ± 0.010e	0.083 ± 0.001c	0.048 ± 0.008b	NDa	0.107 ± 0.006d	NDa	0.110 ± 0.001d	A
v48	2-Phenylacetaldehyde	1637	NDa	0.222 ± 0.004b	NDa	NDa	NDa	NDa	NDa	A
v49	1-Phenylethanone	1645	NDa	NDa	0.039 ± 0.008b	NDa	NDa	NDa	NDa	A
v52	Ethyl 2-phenylacetate	1785	NDa	0.117 ± 0.002c	0.035 ± 0.002b	NDa	NDa	NDa	NDa	A
v54	2-Phenylethanol	>1900	NDa	0.020 ± 0.002b	0.455 ± 0.004e	0.131 ± 0.005d	0.038 ± 0.004c	NDa	NDa	B
***Sulfur-containing volatile compounds***										
v17	(methyldisulfanyl)Methane	1065	NDa	0.114 ± 0.002d	NDa	NDa	0.024 ± 0.004b	0.023 ± 0.004b	0.030 ± 0.002c	A
***Phenol***										
v56	4-Ethenyl-2-methoxyphenol	>1900	NDa	0.021 ± 0.002b	0.020 ± 0.003b	NDa	0.048 ± 0.005c	0.069 ± 0.005d	NDa	B
***Fatty acid metabolism***										
***Fatty Ketones***										
v2	Propan-2-one	812	0.227 ± 0.014b	1.040 ± 0.010f	0.103 ± 0.001a	0.270 ± 0.009c	0.337 ± 0.015e	0.316 ± 0.012d	0.218 ± 0.005b	A
v5	Butan-2-one	901	0.044 ± 0.002b	NDa	0.372 ± 0.008f	0.109 ± 0.006e	0.106 ± 0.006e	0.085 ± 0.006d	0.068 ± 0.001c	A
v12	3-Methylpentan-2-one	1012	NDa	NDa	NDa	NDa	0.175 ± 0.009b	NDa	NDa	A
v23	5-Methylhexan-2-one	1136	NDa	NDa	NDa	NDa	0.02 ± 0.003b	NDa	NDa	A
v27	3-Hydroxy-3-methylbutan-2-one	1239	NDa	NDa	NDa	NDa	NDa	0.078 ± 0.006b	NDa	C
v33	6-Methylhept-5-en-2-one	1334	0.033 ± 0.002a	0.094 ± 0.001e	0.051 ± 0.008bc	0.045 ± 0.002b	0.063 ± 0.005d	0.037 ± 0.005a	0.055 ± 0.001c	A
v34	3-Hydroxypentan-2-one	1337	NDa	NDa	NDa	NDa	0.060 ± 0.005b	0.079 ± 0.006c	NDa	C
v37	Nonan-2-one	1386	NDa	NDa	NDa	NDa	0.043 ± 0.004c	0.012 ± 0.000b	NDa	A
***Fatty alcohols***										
v15	Propan-1-ol	1041	NDa	0.447 ± 0.01c	0.085 ± 0.007b	NDa	NDa	NDa	NDa	A
v24	Butan-1-ol	1150	0.081 ± 0.007c	0.081 ± 0.001c	0.069 ± 0.007b	0.030 ± 0.002a	0.159 ± 0.008e	0.069 ± 0.005b	0.121 ± 0.001d	A
v29	Pentan-1-ol	1254	0.147 ± 0.009cd	0.040 ± 0.002a	0.151 ± 0.005d	0.118 ± 0.005b	0.138 ± 0.007c	0.240 ± 0.010e	0.246 ± 0.006e	A
v32	3-Methylbut-2-en-1-ol	1323	NDa	NDa	NDa	NDa	0.030 ± 0.004b	0.027 ± 0.004b	NDa	C
v35	Hexan-1-ol	1357	0.102 ± 0.011b	0.321 ± 0.007e	0.311 ± 0.002e	0.069 ± 0.003a	0.218 ± 0.011c	0.501 ± 0.016f	0.294 ± 0.007d	A
v36	3-Ethoxypropan-1-ol	1377	NDa	NDa	0.055 ± 0.008b	NDa	NDa	NDa	NDa	A
v39	Oct-1-en-3-ol	1454	0.029 ± 0.002b	0.050 ± 0.002d	0.035 ± 0.002c	0.040 ± 0.002c	0.058 ± 0.005e	0.030 ± 0.004b	NDa	A
v42	2-Ethylhexan-1-ol	1493	NDa	0.044 ± 0.002cd	0.056 ± 0.008e	0.046 ± 0.002d	0.099 ± 0.006f	0.031 ± 0.004b	0.038 ± 0.002bc	A
***Fatty esters***										
v9	Ethyl propanoate	950	NDa	0.240 ± 0.005b	NDa	NDa	NDa	NDa	NDa	A
v14	Ethyl butanoate	1034	NDa	0.271 ± 0.005c	0.251 ± 0.003b	NDa	NDa	NDa	NDa	A
v55	Ethyl tetradecanoate	>1900	NDa	NDa	0.051 ± 0.008b	NDa	NDa	NDa	NDa	B
v57	Ethyl hexadecanoate	>1900	NDa	0.098 ± 0.001b	0.321 ± 0.002c	NDa	NDa	NDa	NDa	B
***Fatty aldehydes***										
v18	Hexanal	1076	1.305 ± 0.086d	0.190 ± 0.003b	0.242 ± 0.003b	0.105 ± 0.004a	0.249 ± 0.012b	0.071 ± 0.005a	0.201 ± 0.004b	A
v31	Octanal	1284	0.026 ± 0.002c	NDa	NDa	0.018 ± 0.001b	0.029 ± 0.004d	NDa	NDa	A
v38	Nonanal	1390	0.265 ± 0.017e	0.182 ± 0.003c	0.215 ± 0.003d	0.192 ± 0.008c	0.193 ± 0.01c	0.051 ± 0.005a	0.080 ± 0.001b	A
v43	Decanal	1496	NDa	0.069 ± 0.002d	0.040 ± 0.008c	NDa	NDa	0.028 ± 0.004b	0.025 ± 0.002b	A
***Fatty carboxylic acid***										
v53	Hexanoic acid	1865	NDa	NDa	NDa	NDa	NDa	NDa	0.075 ± 0.001b	A
***Furans***										
v3	Oxolane	855	0.137 ± 0.008d	NDa	NDa	0.189 ± 0.007e	NDa	0.037 ± 0.005b	0.068 ± 0.001c	A
v26	2-Pentylfuran	1227	0.045 ± 0.002c	0.089 ± 0.001f	0.071 ± 0.007e	0.036 ± 0.002ab	0.062 ± 0.005d	0.029 ± 0.004a	0.040 ± 0.002bc	A
v41	Furan-2-carbaldehyde	1459	0.021 ± 0.002b	NDa	NDa	NDa	NDa	NDa	NDa	A
***Lactone***										
v51	5-Ethyloxolan-2-one	1694	NDa	NDa	0.094 ± 0.007b	NDa	NDa	NDa	NDa	A

^1^ Numbered as in the order of retention indices (RI). ^2^ RI; Retention indices were determined using n-alkanes (C7–C22) as external standards. ^3^ Mean values of relative peak area to that of internal standard ± standard deviation (SD). ^4^ Tentative identification was performed as follows: A, mass spectrum (MS) and retention index (RI) were consistent with those of the mass spectrum database and MS agree with the authentic compound (positive identification, PI); B, MS and PI; C, MS and RI. ^5^ All abbreviations are shown below; AOR (fermented by *Aspergillus oryzae*), ROR (*Rhyzopus oryzae*), SCR (*Sacharomyces cerevisiae*), SFR (*Saccharomycopsis fibuligera*), LFR (*Lactobacillus fermentum*), LPR (*Lactobacillus plantarum*), and RICE (non-fermented rice).^6^ Not detected. ^7^ There are significant differences (*p* < 0.05) among samples fermented by microbes (AOR, ROR, SCR, SFR, LFR, and LPR) and non-fermented rice (RICE) using Duncan’s multiple comparison test between the samples, shown by having different letter in lowercase.

**Table 3:** Distinctive volatile compounds of fermented rice samples.

AOR ^3^			ROR		
No.^1^	*r*-Value ^2^	Volatile Compound	No.	*r*-Value	Volatile Compound
v16	1.00	Ethyl 2-methylbutanoate	v21	1.00	3-Methylbutyl acetate
v48	1.00	2-Phenylacetaldehyde	v51	1.00	5-Ethyloxolan-2-one
v9	1.00	Ethyl propanoate	v36	0.99	3-Ethoxypropan-1-ol
v46	1.00	2-Methylpropanoic acid	v55	0.99	Ethyl tetradecanoate
v6	1.00	2-Methylbutanal	v49	0.98	1-Phenylethanone
v7	1.00	3-Methylbutanal	v54	0.96	2-Phenylethanol
v15	0.98	Propan-1-ol	v57	0.95	Ethyl hexadecanoate
v2	0.97	Propan-2-one	v5	0.95	Butan-2-one
v50	0.97	3-Methylbutanoic acid	v25	0.93	3-Methylbutan-1-ol
v52	0.95	Ethyl 2-phenylacetate	v28	0.75	Styrene
v17	0.95	(methyldisulfanyl)Methane			
v1	0.91	Acetaldehyde			
v10	0.90	Ethyl 2-methylpropanoate			
v33	0.88	6-Methylhept-5-en-2-one			
v43	0.78	Decanal			
v4	0.75	Ethyl acetate			
v29	−0.72	Pentan-1-ol			
**SPR**			**SCR**		
v12	1.00	3-Methylpentan-2-one	v3	0.93	Oxolane
v45	0.99	Butane-2,3-diol			
v23	0.99	5-Methylhexan-2-one			
v37	0.96	Nonan-2-one			
v42	0.93	2-Ethylhexan-1-ol			
v13	0.83	Toluene			
v31	0.82	Octanal			
v11	0.82	Butane-2,3-dione			
v24	0.76	Butan-1-ol			
v22	0.70	1,4-Xylene			
**LFR**			**LPR**		
v30	1.00	3-Hydroxybutan-2-one	v53	1.00	Hexanoic acid
v27	1.00	3-Hydroxy-3-methylbutan-2-one	v40	0.96	Acetic acid
v47	0.83	Butane-1,3-diol			
v56	0.76	4-Ethenyl-2-methoxyphenol			
v34	0.75	3-Hydroxypentan-2-one			
v35	0.74	Hexan-1-ol			

^1^ Numbered as in the order of retention indices. ^2^ The correlation coefficient r was selected as a significant difference (r > 0.7 or r < −0.7) between a microbe and the levels of volatile compounds. ^3^ Sample abbreviations are shown below; AOR (fermented by *Aspergillus oryzae*), ROR (*Rhyzopus oryzae*), SCR (*Saccharomyces cerevisiae*), SFR (*Saccharomycopsis fibuligera*), LFR (*Lactobacillus fermentum*), LPR (*Lactobacillus plantarum*), and RICE (non-fermented rice).

## Supplementary Materials

The following are available online at https://www.mdpi.com/1420-3049/24/11/2123/s1, Table S1: The fold changes of volatile compounds in fermented rice samples (purple boxes indicate newly detected volatile compounds).
