# Supplementary material for: Distinctive Formation of Volatile Compounds in Fermented Rice Inoculated by Different Molds, Yeasts, and Lactic Acid Bacteria
Source: Molecules. 2019 Jun 5;24(11):2123. doi: 10.3390/molecules24112123 (PMC6600562; doi:10.3390/molecules24112123)
Supplement: Supplementary file 1 [file molecules-24-02123-s001.pdf]

Table S1. The fold changes of volatile compounds in fermented rice samples (purple boxes indicate newly detected volatile compounds).

No.[1]	Volatile compound	Fold change[2]					
		AOR[3]	*ROR*	*SCR*	*SFR*	*LFR*	*LPR*
	Carbohydrates metabolism						
	Ethanol fermentation						
v1	Acetaldehyde	0.72	0.30	NC[4]	NC	NC	NC
v4	Ethyl acetate	32.54	25.09	6.83	1.87	3.90	2.33
v8	Ethanol	247.24	398.16	417.43	5.81	8.73	2.08
v40	Acetic acid	0.06	0.15	0.04	0.10	NC	0.48
	Butanediol fermentation						
v11	Butane-2,3-dione	ND[5]	ND	2.61	6.74	3.84	0.88
v30	3-Hydroxybutan-2-one	1.87	7.35	2.91	7.22	331.69	21.29
v45	Butane-2,3-diol	NC	NC	NC	1.66	0.16	NC
v47	Butane-1,3-diol	0.26	NC	NC	0.20	0.51	NC
	Amino acids metabolism						
	Valine degradation						
v10	Ethyl 2-methylpropanoate	0.46	0.20	NC	NC	NC	NC
v19	2-Methylpropan-1-ol	2.44	2.36	0.22	0.03	NC	0.03
v46	2-Methylpropanoic acid	0.37	0.00	NC	NC	NC	NC
	Isoleucine degradation						
v6	2-Methylbutanal	1.48	0.01	0.07	0.01	NC	NC
v16	Ethyl 2-methylbutanoate	0.11	NC	NC	NC	NC	NC
	Leucine degradation						
v7	3-Methylbutanal	334.20	9.40	20.74	10.91	ND	ND
v21	3-Methylbutyl acetate	NC	0.04	NC	NC	NC	NC
v25	3-Methylbutan-1-ol	14.94	39.59	1.30	1.03	0.04	0.08
v50	3-Methylbutanoic acid	1.31	NC	NC	0.32	0.19	NC
	Benzenes						
v13	Toluene	ND	1.44	0.81	2.56	0.80	1.08
v20	Ethylbenzene	ND	2.41	ND	2.66	0.90	1.18
v22	1,4-Xylene	NC	0.05	NC	0.05	NC	NC
v28	Styrene	0.92	2.57	1.96	1.84	1.19	1.43
v44	Benzaldehyde	0.47	0.27	ND	0.60	ND	0.62
v48	2-Phenylacetaldehyde	0.22	NC	NC	NC	NC	NC
v49	1-Phenylethanone	NC	0.04	NC	NC	NC	NC
v52	Ethyl 2-phenylacetate	0.12	0.03	NC	NC	NC	NC
v54	2-Phenylethanol	0.02	0.45	0.13	0.04	NC	NC
	Sulfur-containing volatile compounds						
v17	(methyldisulfanyl)Methane	0.11	NC	NC	0.02	0.02	0.03

Table S1. *Cont.*

			Phenol				
v56	4-Ethenyl-2-methoxyphenol	0.02	0.02	NC	0.05	0.07	NC
			Fatty acids metabolism				
			Fatty ketones				
v2	Propan-2-one	4.59	0.45	1.19	1.48	1.39	0.96
v5	Butan-2-one	ND	8.36	2.45	2.39	1.90	1.52
v12	3-Methylpentan-2-one	NC	NC	NC	0.17	NC	NC
v23	5-Methylhexan-2-one	NC	NC	NC	0.02	NC	NC
v27	3-Hydroxy-3-methylbutan-2-one	NC	NC	NC	NC	0.08	NC
v33	6-Methylhept-5-en-2-one	2.82	1.51	1.36	1.88	1.09	1.65
v34	3-Hydroxypentan-2-one	NC	NC	NC	0.06	0.08	NC
v37	Nonan-2-one	NC	NC	NC	0.04	0.01	NC
			Fatty alcohols				
v15	Propan-1-ol	0.45	0.09	NC	NC	NC	NC
v24	Butan-1-ol	0.99	0.84	0.37	1.96	0.85	1.50
v29	Pentan-1-ol	0.27	1.03	0.80	0.94	1.63	1.68
v32	3-Methylbut-2-en-1-ol	NC	NC	NC	0.03	0.03	NC
v35	Hexan-1-ol	3.15	3.04	0.68	2.14	4.90	2.88
v36	3-Ethoxypropan-1-ol	NC	0.05	NC	NC	NC	NC
v39	Oct-1-en-3-ol	0.04	0.06	0.05	0.10	0.03	0.04
v42	2-Ethylhexan-1-ol	1.73	1.22	1.38	1.99	1.04	ND
			Fatty esters				
v9	Ethyl propanoate	0.24	NC	NC	NC	NC	NC
v14	Ethyl butanoate	0.27	0.25	NC	NC	NC	NC
v55	Ethyl tetradecanoate	NC	0.05	NC	NC	NC	NC
v57	Ethyl hexadecanoate	0.10	0.32	NC	NC	NC	NC
			Fatty aldehydes				
v18	Hexanal	0.15	0.19	0.08	0.19	0.05	0.15
v31	Octanal	ND	ND	0.70	1.14	ND	ND
v38	Nonanal	0.69	0.81	0.73	0.73	0.19	0.30
v43	Decanal	0.07	0.04	NC	NC	0.03	0.03
			Fatty carboxylic acids				
v53	Hexanoic acid	NC	NC	NC	NC	NC	0.08
			Furans				
v3	Oxolane	ND	ND	1.39	ND	0.27	0.49
v26	2-Pentylfuran	1.97	1.57	0.80	1.37	0.65	0.88
v41	Furan-2-carbaldehyde	NC	NC	NC	NC	NC	0.01
			Lactone				
v51	5-Ethyloxolan-2-one	NC	0.09	NC	NC	NC	NC

[1] Numbered as in the order of retention indices (RI).

[2] Log $_2$ fold changes between control (RICE) and fermented samples (AOR, ROR, SCR, SFR, LPR, and LFR) were obtained. Newly detected volatile compounds were calculated by adding Constance 1 to each value.

[3] All abbreviations are shown below; AOR (fermented by *Aspergillus oryzae*), ROR (*Rhyzopus oryzae*), SCR (*Sacharomyces cerevisiae*), SFR (*Saccharomycopsis fibuligera*), LFR (*Lactobacillus fermentum*), and LPR (*Lactobacillus plantarum*).

[4] Not changed

[5] Not detected
